# Neuroretinal Layer Thinning on OCT Imaging and Hemoglobin A_1c_ in Youth With Type 1 Diabetes

**DOI:** 10.1001/jamaophthalmol.2026.1341

**Published:** 2026-06-25

**Authors:** Supriya Ramanujam, Roomasa Channa, T. Y. Alvin Liu, Elizabeth A. Brown, Neha Parimi, Yoohee Claire Kim, Lee Bromberger, Harold P. Lehmann, Michael D. Abramoff, Risa M. Wolf

**Affiliations:** 1 Division of Endocrinology, Department of Pediatrics, Johns Hopkins School of Medicine, Baltimore, Maryland; 2 Department of Ophthalmology and Visual Sciences, University of Wisconsin, Madison; 3 Wilmer Eye Institute at the Johns Hopkins School of Medicine, Baltimore, Maryland; 4 Section on Biomedical Informatics and Data Science, Johns Hopkins University, Baltimore, Maryland; 5 Department of Ophthalmology and Visual Sciences, The University of Iowa, Iowa City; 6 Digital Diagnostics, Coralville, Iowa; 7 Iowa City VA Medical Center, Iowa City, Iowa; 8 Department of Biomedical Engineering, The University of Iowa, Iowa City; 9 Department of Electrical and Computer Engineering, The University of Iowa, Iowa City

## Abstract

**Importance:**

Diabetic retinal neurodegeneration precedes vascular changes associated with diabetic retinal disease (DRD). Studies in adults with type 1 diabetes (T1D) show there is retinal layer thinning with DRD, yet there are limited data in youth with T1D.

**Objectives:**

To determine if retinal layer thickness changes on optical coherence tomography (OCT) imaging were associated with glycemic outcomes and DRD in youth.

**Design, Setting, and Participants:**

This prospective cohort study was conducted at an academic pediatric diabetes center among youth with T1D aged 9 to 21 years participating in the ACCESS2 (AI for Pediatric Diabetic Eye Exams Study 2) study. Participants were enrolled and data were collected July 11, 2022, and April 30, 2025. Data analysis was performed from June 2025 through October 2025.

**Exposure:**

OCT imaging.

**Main Outcomes and Measures:**

The primary outcome was macular OCT volumes, which were segmented by the Topcon Maestro camera software and reviewed by the Wisconsin Reading Center for 3 neuroretinal layers: (1) retinal nerve fiber layer (RNFL) thickness, (2) ganglion cell and inner plexiform layer (GCL+IPL) thickness, and (3) GCL+IPL+RNFL thickness, as well as total retinal thickness. Layer thicknesses were analyzed for associations with glycemic outcomes and DRD and for potential covariates.

**Results:**

A total of 294 youth with T1D (n = 578 eyes), among whom mean (SD) age was 15.8 (2.8) years, 153 participants (52.0%) were female, and 108 participants (36.7%) had public insurance, were included. Participants had a median (IQR) duration of diabetes of 7.0 (4.6-10.1) years and a median (IQR) hemoglobin A_1c_ (HbA_1c_) of 8.5% (7.5%-9.9%); 210 participants (71.4%) used an insulin pump. Of the total 578 eyes, 65 eyes (11.2%) had mild DRD and 10 eyes (1.73%) had moderate DRD. In adjusted analyses, moderate DRD vs no DRD was associated with RNFL thickness of −1.2 µm (95% CI, −2.9 to 0.5; *P* = .20), GCL+IPL thickness of −1.2 µm (95% CI, −2.8 to 0.4; *P* = .19), and outer retinal layer thickness of −0.8 µm (95% CI, −3.9 to 2.2; *P* = .80). In multivariable models, GCL+IPL and outer retinal layer thickness were associated with HbA_1c_ (β = −0.39; 95% CI, −0.78 to −0.01; *P* = .04; and β = −0.81; 95% CI, −1.49 to −0.12; *P* = .02, respectively).

**Conclusions and Relevance:**

In this prospective cohort study, neuroretinal layer thinning was observed in youth with T1D without clinically apparent DRD and was associated with higher HbA_1c_. These findings support elucidating the development of diabetic retinal neurodegeneration and its potential role as a biomarker of retinal vascular disease in youth.

## Key Points

**Question:**

Are retinal layer thickness changes on optical coherence tomography (OCT) imaging in youth with type 1 diabetes associated with glycemic outcomes and diabetic retinopathy?

**Finding:**

In this cohort study, thinning of the ganglion cell and inner plexiform layer (GCL+IPL) and outer retinal layers was associated with higher hemoglobin A_1c_ (HbA_1c_). There was no difference in total retinal thickness, retinal nerve fiber layer thickness, GCL+IPL thickness, or outer retinal layer thickness by presence or absence of diabetic retinopathy or severity of retinopathy.

**Meaning:**

Neuroretinal layer thinning was observed in youth with type 1 diabetes without clinically apparent retinopathy and was associated with higher HbA_1c_.

## Teaser

This prospective cohort study determines if retinal layer thickness changes on optical coherence tomography (OCT) imaging were associated with glycemic outcomes and diabetic retinal disease in youth with type 1 diabetes.

## Introduction

Diabetic retinal disease (DRD) is a complication of diabetes that leads to vision loss.^1^ The SEARCH and TODAY2 studies showed that 52% of youth with type 1 diabetes (T1D) and 49% to 55% of youth with type 2 diabetes (T2D) had DRD.^2,3^ Risk factors for DRD include longer duration of diabetes, higher hemoglobin A_1c_ (HbA_1c_), and hypertension.^4,5,6^ Moreover, DRD risk was found to increase 20% to 30% for every 1-point increase in HbA_1c_ value.^7,8^ While DRD is vision threatening, early diagnosis and treatment of DRD is 90% effective at preventing vision loss, thus highlighting the importance of early detection.^9^

While DRD is classically characterized by vascular changes on fundus imaging, newer evidence shows that diabetic retinal neurodegeneration (DRN)—damage to the neuroretinal tissue or thinning of the retina—is associated with the earliest stages of DRD and precedes any macroscopic or microscopic vascular changes.^10,11^ A 2023 review of evidence on biomarkers of DRD suggested that DRN be incorporated into the DRD classification framework as an early marker of retinopathy.^12^ Ocular changes in the neuroretina have been detected in pediatric cohorts of patients with neurological abnormalities using optical coherence tomography (OCT).^13,14,15,16^ OCT produces high-resolution, micron-level, 3-dimensional images of the retina in a noninvasive manner and can be used to measure neuroretinal thickness.^17^

Studies have used OCT imaging to analyze total neuroretinal thickness, as well as the thickness of individual retinal layers in people with diabetes. Total neuroretinal thickness has been found to be lower in adults with diabetes, lower in adults with T1D and DRD compared with those without DRD, and associated with diabetes duration.^11,18,19^ In a pediatric cohort of youth with T2D, the TODAY2 study found reductions in total neuroretinal thickness with increasing duration of diabetes compared with baseline measurements.^20^ Several small studies have evaluated OCT imaging in youth with T1D, predominantly in youth with a short duration of diabetes and in those without DRD with inconsistent findings on changes in retinal thickness.^21,22^ In a 2025 pilot study using OCT-angiography in youth with T1D and T2D, we demonstrated that choroidal vascularity index, choroidal vascular volume, and choriocapillaris thickness were higher in youth with T1D compared with controls.^23^ There are limited data regarding the neural retinal thickness of pediatric patients with T1D, as well as potential associations with glycemic outcomes, DRD presence and severity, and use of diabetes technology in the current era.

We hypothesize that neuroretinal thinning is associated with higher HbA_1c_, longer duration of diabetes, and greater DRD severity in youth with T1D. This study aims to use OCT to examine associations between neuroretinal changes and HbA_1c_, DRD and severity, race and ethnicity, diabetes duration, and use of diabetes technology in youth with T1D.

## Methods

### Trial Design

This prospective cohort study was conducted as a part of the larger ACCESS2 (AI for Pediatric Diabetic Eye Exams Study 2) study (NCT05463289) at the Johns Hopkins Pediatric Diabetes Center. Participants were enrolled at 2 locations (Johns Hopkins Hospital and Mount Washington Pediatric Hospital) between July 11, 2022, and April 30, 2025. The study complied with the Declaration of Helinski and the Johns Hopkins institutional review board granted approval. Patients provided written consent and were offered compensation for their participation. The study followed the Strengthening the Reporting of Observational Studies in Epidemiology (STROBE) reporting guidelines.

### Participants

Of the ACCESS2 study cohort, this substudy analyzed interpretable OCT volumes from 286 enrolled youth with T1D aged 9 to 21 years. As in the ACCESS2 study, eligible participants met the American Diabetes Association’s criteria for need of a diabetic retinopathy screening (diabetes duration ≥3 years and age greater than 11 years or in puberty), did not have known DRD, and had not had a diabetic eye examination in the past year. An enriched cohort of 15 participants with T1D who either had known DRD or who were deemed to be at high risk for DRD was also included. Participants with uninterpretable OCT images were excluded.

### Study Procedures

Participants underwent fundus photography with the Topcon NW400 (Topcon Healthcare) with LumineticsCore AI technology (Luminetics Core, formerly IDx-DR) and OCT imaging using the Topcon Maestro spectral-domain OCT camera (Topcon Healthcare). For OCT imaging, 1 volume scan was taken of each eye and automatically segmented into 9 sectors (center, outer superior, inner superior, inner inferior, outer inferior, outer temporal, inner temporal, inner nasal, and outer nasal). A 6 × 6-mm 3-dimensional macular cube (512 × 128 sampling density) was acquired. Pharmacological dilation was not required.

Using the OCT software, mean thickness for the retinal nerve fiber layer (RNFL), ganglion cell and inner plexiform layer (GCL+IPL), and GCL+IPL+RNFL layers of the retina, as well as total retinal thickness, was obtained. Outer retinal layer thickness was calculated as total retinal thickness without GCL+IPL+RNFL thickness (eFigure 1 in Supplement 1).

Fundus and OCT images were sent to the Wisconsin Reading Center for analysis by trained readers. Fundus images were graded using the International Clinical Diabetic Retinopathy Scale classification to determine presence and stage of DRD and diabetic macular edema. For patient-level descriptive analyses, a patient was classified as positive for DRD if it was present in either eye, and DRD stage was based on the most severely affected eye. Eye-level analyses assigned DRD independently for each eye. OCT images were evaluated for segmentation accuracy and analyzed for the presence of intraretinal fluid, subretinal fluid, vitreomacular traction center subfield, and macular holes. Eyes with definite intraretinal or subretinal fluid were excluded from analysis due to distortions in retinal layers. Wisconsin Reading Center quality assessments of adequate and Maestro quality score greater than or equal to 40 were used to determine if OCT readings were sufficient to analyze. Maestro camera retinal thickness measurements were used for the analysis itself. If the image of 1 eye was deemed to be of inadequate quality, the other eye was still included in analysis. All data were entered into REDCap by an analyst and verified by a second person.^24,25^ Participant demographic and clinical data was obtained from the electronic health record.

### Outcomes Measured

The primary outcomes were associations of RNFL and GCL+IPL thickness with HbA_1c_ and DRD. Secondary outcomes included outer retinal layer thickness (total retinal thickness without GCL+IPL+RNFL thickness) and associations with diabetes duration, race and ethnicity, and diabetes technology usage.

### Statistical Analysis

Descriptive statistics were reported using frequency and percentage for categorical variables; skewed continuous variables were described using median and interquartile range, and normally distributed variables using mean and standard deviation. For each participant, the 5 most recent HbA_1c_ measurements were averaged, and the average HbA_1c_ was used in all analysis. Analysis was performed to compare participants by HbA_1c_ greater or less than 8% via Pearson χ^2^ tests and Fisher exact tests for categorical variables and via 2-sample *t* tests and Wilcoxon rank sum tests for continuous variables. Participants were categorized using an HbA_1c_ threshold of 8%, as prior pediatric studies have demonstrated increased risk of diabetic retinopathy among patients with HbA_1c_ greater than 8%.^26^ (To convert HbA_1c_ from percentage of total hemoglobin to proportion of total hemoglobin, multiply by 0.01.) RNFL thickness, GCL+IPL thickness, and total retinal thickness were determined by Maestro Topcon camera readout. The 9 sector readings were averaged to determine a single reading for each eye. Outer retinal layer thickness was calculated by subtracting the RNFL and GCL+IPL thickness from total retinal thickness.

For OCT-specific variables, adjusted means, 95% confidence intervals, and *P* values were estimated using a linear mixed-effects model that accounted for within-participant correlation due to measurements from both eyes. Least-squares means were estimated for DRD categories, and pairwise differences were tested using Tukey adjustment.

To assess our hypothesis that neuroretinal thinning is associated with higher HbA_1c_, longer diabetes duration, and DRD presence, a multivariable mixed-effects regression model was used to evaluate associations between duration of diabetes, HbA_1c_, and retinal thickness. Separate models were created to predict RNFL, GCL+IPL, and outer retinal layer thicknesses. Other covariates include sex, race and ethnicity, age, insurance type, DRD severity, insulin delivery method, site, and total retinal thickness without RNFL for the RNFL model and without the entire ganglion cell complex for the GCL+IPL model. Candidate cardiovascular covariates were evaluated during model development but were not retained because they lacked association to the outcomes and did not materially alter effect estimates. Separate models for the subset of participants using a continuous glucose monitor (CGM) were created to evaluate associations with percentage time in range (TIR). To account for the nonindependence of observations arising from the inclusion of data from both eyes, a random intercept for each individual was included in the model to account for within-participant correlation. Models were adjusted for total retinal thickness^11^ (eTables 4-7 in Supplement), and formal interaction tests were run between race and HbA_1c_. SAS software version 9.4 (SAS Institute) was used to perform these analyses; *P* values were 2-sided and were not adjusted for multiple analyses. *P* < .05 was considered significant.

## Results

### Participant Demographics and Clinical Characteristics

A total cohort of 294 youth with T1D were included, and 284 of 294 had OCT images of sufficient quality for interpretation in both eyes, while the remaining 10 had OCT images of sufficient quality in 1 eye. A total of 578 eyes were available for interpretation. Consensus DRD interpretation was also available for 293 of 294 participants (eFigure 2 in Supplement 1). As shown in Table 1, this cohort had a mean (SD) age of 15.8 (2.8) years, 153 participants (52.0%) were female, and 108 participants (36.7%) had public insurance. For electronic health record–derived race and ethnicity, 2 participants (0.7%) were Asian, 10 (3.4%) were Hispanic, 20 (6.8%) were multiracial, 79 (26.9%) were non-Hispanic Black, and 183 (62.2%) were non-Hispanic White. Participants had a median (IQR) duration of diabetes of 7.0 (4.6-10.1) years and a median (IQR) HbA_1c_ of 8.5% (7.5%-9.9%). In this cohort, 257 participants (87.4%) were prescribed a CGM and 210 (71.4%) used an insulin pump. Of 293 participants with consensus DRD grading, 228 participants (77.8%) had no apparent DRD, 55 (18.8%) had mild NPDR, and 10 (3.4%) had moderate DRD. Of the total 578 eyes, 65 eyes (11.2%) had mild DRD and 10 eyes (1.73%) had moderate DRD. Participants with an HbA_1c_ greater than 8% were more likely to be non-Hispanic Black (39.3% vs 6.3%; *P* < .001), have Medicaid insurance (48.6% vs 17.1%; *P* < .001), have a lower TIR on CGM (40.0% vs 59.0%; *P* < .001), and have a higher risk of mild or greater DRD (27.9% vs 12.7%; *P* = .006). Participants with retinopathy were more likely to be non-Hispanic Black (44.6% vs 21.9%; *P* = .008), have public insurance (49.2% vs 33.3%; *P* = .02), have a longer duration of diabetes (9 vs 6.5 years; *P* < .001) and higher HbA_1c_ (9.4% vs 8.3%; *P* < .001), and, for those using CGM, have a lower percentage time in target range (42.4% vs 50%; *P* = .004) and greater percentage time above target glucose range (55.9% vs 48.0%; *P* = .009).

**Table 1.** Descriptive Characteristics of Participants Undergoing Optical Coherence Tomography Imaging

Characteristic	No. (%)			Difference (95% CI)^a^	*P* value
	HbA_1c_ ≤8% (n = 111)	HbA_1c_ >8% (n = 183)	Total (n = 294)		
Demographics					
Sex at birth					
Female	60 (54.1)	93 (50.8)	153 (52.0)	−3.2% (−8.5% to 14.9%)	.59^b^
Male	51 (45.9)	90 (49.2)	141 (48.0)	3.2% (−14.9% to 8.5%)	
Race and ethnicity^c^					
Asian	1 (0.9)	1 (0.5)	2 (0.7)	0.4% (−2.2% to 4.4%)	<.001^d^
Hispanic	1 (0.9)	9 (4.9)	10 (3.4)	−4.0% (−8.3% to 0.4%)	
Multiracial^e^	4 (3.6)	16 (8.7)	20 (6.8)	−5.1% (−10.8% to 1.0%)	
Non-Hispanic Black	7 (6.3)	72 (39.3)	79 (26.9)	−33.0% (−41.2% to −24.2%)	
Non-Hispanic White	98 (88.3)	85 (46.4)	183 (62.2)	41.8% (31.8% to 50.7%)	
Age, mean (SD), y	15.7 (2.89)	15.8 (2.72)	15.8 (2.78)	−0.2 (−0.8 to 0.5)	.60^f^
Medicaid insurance	19 (17.1)	89 (48.6)	108 (36.7)	−31.5% (−41.1% to −20.9%)	<.001^b^
Income >$50 000	83 (88.3)	103 (70.1)	186 (77.2)	18.2% (7.8% to 27.8%)	.001^b^
Missing, No.	17	36	53	NA	NA
Maximum parent education includes more than a high school diploma	90 (83.3)	97 (55.4)	187 (66.1)	27.9% (17.2% to 37.6%)	<.001^b^
Missing, No.	3	8	11	NA	NA
Diabetes					
Duration of T1D, median (IQR), y	6.9 (4.0 to 9.6)	7.1 (4.7 to 10.5)	7.0 (4.6 to 10.1)	−0.4 (−1.2 to 0.4)	.30^g^
HbA_1c_, mean of last 5 values, median (IQR), %	7.3 (6.9 to 7.6)	9.4 (8.6 to 11.0)	8.5 (7.5 to 9.9)	−2.3 (−2.6 to −2.0)	<.001^g^
DRD severity in worse eye					
No apparent retinopathy	96 (87.3)	132 (72.1)	228 (77.8)	14.4% (4.8% to 23.2%)	.006^d^
Mild nonproliferative DRD	13 (11.8)	42 (23.0)	55 (18.8)	−11.2% (−19.6% to −2.2%)	
Moderate nonproliferative DRD	1 (0.9)	9 (4.9)	10 (3.4)	−4.1% (−8.3% to 0.4%)	
Missing, No.	1	0	1	NA	
Glycemia					
Continuous glucose monitor use	106 (95.5)	151 (82.5)	257 (87.4)	13.0% (5.8% to 19.9%)	.001^b^
Time in target range (70-180 mg/dL), median (IQR), %	59.0 (50.0 to 66.0)	40.0 (23.2 to 50.1)	48.8 (36.0 to 60.0)	20.4 (16.0 to 24.8)	<.001^g^
Time above range (>180 mg/dL), median (IQR), %	39.0 (31.0 to 49.0)	57.0 (48.0 to 75.0)	50.0 (38.0 to 61.0)	−20.3 (−24.5 to −16.0)	<.001^g^
Time below range (<70 mg/dL), median (IQR), %	1.0 (0.1 to 2.0)	1.0 (0.0 to 2.0)	1.0 (0.0 to 2.0)	0.15 (0.0 to 0.3)	.49^g^
Insulin pump use	102 (91.9)	108 (59.0)	210 (71.4)	32.9% (23.6% to 41.3%)	<.001^b^
Other metabolic health					
Systolic blood pressure, mean (SD), mm Hg	114.7 (8.67)	118.5 (8.99)	117.1 (9.05)	−3.9 (−6.0 to −1.8)	<.001^f^
Diastolic blood pressure, mean (SD), mm Hg	69.3 (6.91)	70.5 (7.25)	70.0 (7.14)	−1.2 (−2.9 to 0.5)	.17^f^
BMI *z* score, median (IQR)	0.8 (0.3 to 1.2)	0.9 (0.3 to 1.5)	0.9 (0.3 to 1.4)	−0.1 (−0.4 to 0.1)	.19^g^
Total cholesterol, median (IQR), mg/dL	169.0 (150.0 to 186.0)	175.0 (156.0 to 197.0)	172.0 (151.0 to 195.0)	−5.0 (−13.0 to 3.0)	.21^g^
Low-density lipoprotein cholesterol, median (IQR), mg/dL	92.5 (79.0 to 110.0)	100.0 (80.0 to 118.0)	95.0 (80.0 to 114.0)	−3.5 (−10.0 to 3.0)	.27^g^
Triglycerides, median (IQR), mg/dL	74.0 (57.0 to 97.0)	85.0 (64.0 to 120.0)	80.0 (60.0 to 110.0)	−12.5 (−21.0 to −4.0)	.005^g^
Site of visit					
Downtown site	52 (46.8)	104 (56.8)	156 (53.1)	−10% (−21.5% to 1.8%)	.10^b^
Suburban site	59 (53.2)	79 (43.2)	138 (46.9)	10% (−1.8% to 21.5%)	

Abbreviations: BMI, body mass index (calculated as weight in kilograms divided by height in meters squared); DRD, diabetic retinal disease; HbA_1c_, hemoglobin A_1c_; NA, not applicable; T1D, type 1 diabetes.

SI conversion factors: To convert glucose from mg/dL to mmol/L, multiply by 0.0555; HbA_1c_, from percentage to proportion of total hemoglobin, multiply by 0.01; low-density lipoprotein cholesterol and total cholesterol, from mg/dL to mmol/L, multiply by 0.0259; triglycerides, from mg/dL to mmol/L, multiply by 0.0113.

^a^ Confidence intervals: categorical variables use score confidence limits; normally distributed variables use *t* intervals for the mean difference; and skewed variables use Hodges-Lehmann estimates of median difference.

^b^ χ^2^ *P* value.

^c^ Obtained from the electronic health record.

^d^ Fisher exact *P* value.

^e^ Multiracial participants included 10 participants of Black and White race; 5 participants of Asian and White race; 1 participant of Native Hawaiian or Other Pacific Islander and other race; 1 participant of American Indian or Alaska Native, Black, and White race; and 1 participant of Black, White, and other race.

^f^ Equal variance 2-sample *t* test.

^g^ Wilcoxon rank sum *P* value.

### Measurements of Retinal Thickness

As shown in Table 2, the mean total retinal thickness was 282.65 µm (95% CI, 280.89-284.42), the mean RNFL thickness was 27.83 µm (95% CI, 27.53-28.12), the mean GCL+IPL thickness was 76.98 µm (95% CI, 76.29-77.66), and the mean outer retinal layer thickness was 177.95 µm (95% CI, 176.72-179.18). Differences in measurements of total retinal thickness, RNFL thickness, GCL+IPL thickness, and outer retinal layer thickness by degree of DRD in this youth cohort with T1D were small. In adjusted analyses, moderate DRD vs no DRD was associated with RNFL thickness of −1.2 µm (95% CI, −2.9 to 0.5; *P* = .20), GCL+IPL thickness of −1.2 µm (95% CI, −2.8 to 0.4; *P* = .19), and outer retinal layer thickness of −0.8 µm (95% CI, −3.9 to 2.2; *P* = .80). Mild DRD vs no DRD was associated with RNFL thickness of 0.4 µm (95% CI, −0.2 to 0.9; *P* = .39), GCL+IPL thickness of 0.4 µm (95% CI, −0.2 to 0.9; *P* = .41), and outer retinal layer thickness of −0.2 µm (95% CI, −1.5 to 1.0; *P* = .90).

**Table 2.** Retinal Thickness Measurements of Participants Undergoing Optical Coherence Tomography (OCT) Imaging

OCT retinal thickness measurements	Presence of diabetic retinopathy, mean (95% CI), µm^a^				*P* value
	Total (n = 578 eyes)	No apparent retinopathy (n = 500 eyes)	Mild DRD (n = 65 eyes)	Moderate DRD (n = 10 eyes)	
Total retinal thickness	282.65 (280.89-284.42)	282.64 (280.82-284.45)	282.85 (280.64-285.05)	280.36 (276.50-284.23)	.38
RNFL	27.83 (27.53-28.12)	27.80 (27.49-28.10)	28.17 (27.58-28.76)	26.60 (25.22-27.98)	.07
GCL+IPL	76.98 (76.29-77.66)	76.96 (76.25-77.66)	77.31 (76.46-78.17)	75.75 (74.25-77.25)	.06
Outer retinal layers	177.95 (176.72-179.18)	177.97 (176.71-179.24)	177.75 (176.19-179.30)	177.15 (176.19-179.30)	.78

Abbreviations: DRD, diabetic retinal disease; GCL+IPL, ganglion cell and inner plexiform layer; RNFL, retinal nerve fiber layer.

^a^ Least-square means, confidence intervals, and *P* values are derived from a linear mixed model, adjusting for within-participant correlation; *P* values test the null hypothesis that the group means are equal across all 3 groups. A small value suggests at least 1 group differs. *P* values calculated for 575 eyes, excluding 3 eyes without DRD consensus that are included in the total category.

### Association of Retinal Layer Thickness With Clinical Characteristics

The RNFL, GCL+IPL, and the outer retinal layers (total retinal thickness other than the ganglion cell complex), which together compose the entire retinal thickness, were investigated in univariate and multivariable analysis. In unadjusted analysis, Black race, Medicaid insurance, higher HbA_1c_, and use of injections as the insulin delivery method were associated with thinner measurements in all 3 layers (RNFL in Table 3, GCL+IPL in Table 4, and outer retinal layers in Table 5; eTables 1 and 2 and eFigure 3 in Supplement 1). Female sex was associated with lower thickness of the GCL+IPL and outer retinal layers (Tables 4 and 5). Lower thickness of the outer retinal layers was also associated with multiracial identity, a shorter duration of diabetes, screening at the downtown site, and a lower time in target range for those using CGM (Table 5; eTable 3 in Supplement 1).

**Table 3.** Linear Mixed-Effect Model for Retinal Nerve Fiber Layer (RNFL) Thickness (µm) Accounting for Within-Participant Correlation (n = 575 Eyes)

Effect	Unadjusted		Adjusted	
	Estimate (95% CI)	*P* value	Estimate (95% CI)	*P* value
Intercept	NA	NA	17.70 (11.63 to 23.76)	<.001
Sex female	−0.55 (−1.14 to 0.04)	.07	−0.40 (−0.97 to 0.17)	.17
Race (reference = White)		.002		.33
Asian	−2.03 (−5.53 to 1.46)	.25	−1.11 (−4.53 to 2.31)	.52
Black	−1.36 (−2.03 to −0.70)	<.001	−0.84 (−1.64 to −0.04)	.04
Hispanic	−0.80 (−2.41 to 0.81)	.33	−0.46 (−2.09 to 1.17)	.58
Multiracial	−0.44 (−1.60 to 0.72)	.45	−0.17 (−1.30 to 0.96)	.77
Age	0.01 (−0.09 to 0.12)	.80	0.09 (−0.02 to 0.21)	.12
Medicaid insurance	−0.84 (−1.45 to −0.24)	.006	−0.30 (−0.94 to 0.35)	.37
Duration of diabetes	−0.03 (−0.11 to 0.05)	.47	−0.07 (−0.15 to 0.02)	.14
DRD severity (reference = no apparent retinopathy)		.06		.06
Mild DRD	0.38 (−0.19 to 0.94)	.19	0.52 (−0.06 to 1.09)	.08
Moderate DRD	−1.20 (−2.56 to 0.16)	.08	−0.89 (−2.26 to 0.49)	.21
Hemoglobin A_1c_ (mean over 5 most recent visits)	−0.18 (−0.33 to −0.04)	.01	0.03 (−0.15 to 0.20)	.77
Insulin injections (reference = pump)	−0.89 (−1.53 to −0.24)	.007	−0.49(−1.24 to 0.25)	.19
Total retinal thickness without RNFL (outer retinal layers)	0.05 (0.03 to 0.06)	<.001	0.04 (0.02 to 0.06)	.001
Downtown site (reference = suburban)	0.13 (−0.46 to 0.72)	.67	0.44 (−0.17 to 1.05)	.16

Abbreviations: DRD, diabetic retinal disease; NA, not applicable.

**Table 4.** Linear Mixed-Effect Model for Ganglion Cell and Inner Plexiform Layer (GCL+IPL) Thickness (µm) Accounting for Within-Participant Correlation (n = 575 Eyes)

Effect	Unadjusted		Adjusted	
	Estimate (95% CI)	*P* value	Estimate (95% CI)	*P* value
Intercept	NA	NA	52.06 (42.17 to 61.96)	<.001
Sex female	−1.43 (−2.80 to −0.06)	.04	−1.20 (−2.47 to 0.07)	.06
Race (reference = White)		.08		.14
Asian	−7.60 (−15.92 to 0.72)	.07	−5.62 (−13.27 to 2.02)	.15
Black	−1.83 (−3.41 to −0.25)	.02	1.66 (−0.13 to 3.45)	.07
Hispanic	−0.83 (−4.63 to 2.98)	.67	1.43 (−2.21 to 5.07)	.44
Multiracial	0.41 (−2.35 to 3.16)	.77	2.01 (−0.53 to 4.56)	.12
Age	−0.0002 (−2.35 to 3.16)	.99	−0.13 (−0.39 to 0.13)	.32
Medicaid insurance	−2.01 (−3.43 to −0.60)	.005	−1.33 (−2.77 to 0.11)	.07
Duration of diabetes	0.16 (−0.02 to 0.35)	.08	0.14 (−0.05 to 0.33)	.16
DRD severity (reference = no apparent retinopathy)		.05		.06
Mild DRD	0.36 (−0.19 to 0.91)	.20	0.43 (−0.11 to 0.96)	.12
Moderate DRD	−1.20 (−2.54 to 0.14)	.08	−0.97 (−2.28 to 0.35)	.15
Hemoglobin A_1c_ (mean over 5 most recent visits)	−0.62 (−0.96 to −0.29)	<.001	−0.39 (−0.78 to −0.01)	.04
Insulin injections (reference = pump)	−2.01 (−3.52 to −0.50)	.009	−0.77 (−2.45 to 0.90)	.36
Total retinal thickness without GCL+IPL+RNFL	0.18 (0.14 to 0.22)	<.001	0.17 (0.12 to 0.21)	<.001
Downtown site (reference = suburban)	0.89 (−0.49 to 2.27)	.20	0.03 (−1.33 to 1.39)	.97

Abbreviations: DRD, diabetic retinal disease; NA, not applicable; RNFL, retinal nerve fiber layer.

**Table 5.** Linear Mixed-Effect Model for Total Retinal Thickness Without Ganglion Cell, Inner Plexiform Layer, and Retinal Nerve Fiber Layer (µm) Accounting for Within-Participant Correlation (n = 575 Eyes)

Effect	Unadjusted		Adjusted	
	Estimate (95% CI)	*P* value	Estimate (95% CI)	*P* value
Intercept	NA	NA	183.36 (178.33 to 196.98)	<.001
Sex female	−3.05 (−5.51 to −0.60)	.02	−2.97 (−5.23 to −0.71)	.01
Race (reference = White)		<.001		<.001
Asian	−13.10 (−26.94 to 0.75)	.06	−11.74 (−25.39 to 1.91)	.09
Black	−9.69 (−12.31 to −7.06)	<.001	−7.62 (−10.77 to −4.48)	<.001
Hispanic	−2.70 (−9.04 to 3.63)	.40	−0.70 (−7.21 to 5.82)	.83
Multiracial	−5.05 (−9.64 to −0.47)	.03	−3.86 (−8.40 to 0.68)	.10
Age	0.14 (−0.31 to 0.58)	.55	0.04 (−0.42 to 0.51)	.86
Medicaid insurance	−4.25 (−6.77 to −1.74)	.001	−0.94 (−3.52 to 1.65)	.48
Duration of diabetes	0.35 (0.02 to 0.68)	.04	0.27 (−0.08 to 0.61)	.13
DRD severity (reference = no apparent retinopathy)		.78		.97
Mild DRD	−0.23 (−1.25 to 0.80)	.67	−0.01 (−1.04 to 1.02)	.98
Moderate DRD	−0.82 (−3.33 to 1.69)	.52	−0.33 (−2.84 to 2.17)	.79
Hemoglobin A_1c_ (mean over 5 most recent visits)	−1.48 (−2.08 to −0.89)	<.001	−0.81 (−1.49 to −0.12)	.02
Insulin injections (reference = pump)	−3.76 (−6.46 to −1.05)	.007	0.94 (−2.05 to 3.92)	.54
Downtown site (reference = suburban)	−4.13 (−6.57 to −1.70)	.001	−2.15 (−4.58 to 0.29)	.08

Abbreviations: DRD, diabetic retinal disease; NA, not applicable.

Multivariable analysis was conducted to adjust for confounding. All models adjusted for sex, race and ethnicity, age, insurance type, DRD stage, mean HbA_1c_, insulin delivery method, and location of screening. The model for the RNFL adjusts for total retinal thickness other than RNFL, and the GCL+IPL model adjusts for outer retinal layer thickness. In adjusted analysis, RNFL thickness was associated with Black race (β = −0.84 µm; 95% CI, −1.64 to −0.04; *P* = .04) and total retinal thickness without RNFL (β = 0.04 µm; 95% CI, 0.02-0.06; *P* = .001) (Table 3). GCL+IPL thickness was associated with outer retinal thickness (β = 0.17 µm; 95% CI, 0.12-0.21; *P* < .001) and HbA_1c_ (β = −0.39; 95% CI, −0.78 to −0.01; *P* = .04). For each 1-unit increase in HbA_1c_, there was an associated 0.39 µm lower GCL+IPL thickness (Table 4). The thickness of the outer retinal layers was associated with female sex (β = −2.97; 95% CI, −5.23 to −0.71; *P* = .01), Black race (β = −7.62; 95% CI, −10.77 to −4.48; *P* < .001), and HbA_1c_ (β = −0.81; 95% CI, −1.49 to −0.12; *P* = .02). Each 1-unit increase in HbA_1c_ was associated with 0.81 µm lower thickness in the outer retinal layer (Table 5). For the subset of participants using CGM, multivariable linear mixed models showed that outer retinal layer thickness was also directly associated with percentage time in target range (β = 0.08 µm; 95% CI, 0.01-0.15; *P* = .03) (eTable 3 in Supplement 1).

## Discussion

In this study of a diverse cohort of youth with T1D in the current age of diabetes technologies, we found retinal layer thickness on OCT imaging to be associated with HbA_1c_ and not with diabetes duration or DRD presence after adjusting for other factors. Thinning of the GCL+IPL and outer retinal layers was associated with a higher HbA_1c_ level, and similarly, participants spending more time in the target range of 70 to 180 mg/dL had greater thickness in outer retinal layers. (To convert glucose from mg/dL to mmol/L, multiply by 0.0555.) To our knowledge, this is the first analysis of retinal thickness using OCT in a large cohort of youth with T1D of considerable duration and including participants with retinopathy.

Similar to other studies in children, we found retinal layer thinning was associated with higher HbA_1c_.^11,19,27,28,29^ In unadjusted analysis, there was thinning of RNFL, GCL+IPL, and outer retinal layers with higher HbA_1c_, and similarly with use of insulin injections compared with insulin pumps. Use of insulin pumps was associated with lower HbA_1c_ levels, which likely explains the association of retinal layer thinning with insulin injections.^30^ Even after adjusting for other factors in multivariable models, GCL+IPL and outer retinal layer thinning was found to be associated with higher HbA_1c_. We previously showed in a prospective study in adults with T1D that neuroretinal thinning developed over 4 years and was associated with duration of diabetes, but not with HbA_1c_ or DRD presence.^19^ However, similar to our current findings, pediatric studies in Turkey and Poland found that HbA_1c_ was inversely related to RNFL thickness.^28,29^ Furthermore, the UK Biobank study similarly found that HbA_1c_ and GCL+IPL thickness were inversely correlated in people with diabetes, although its population differed in its adult cohort, higher socioeconomic status, and limited diversity.^11^ The UK Biobank study demonstrated GCL+IPL thinning of −0.26 microns per unit of HbA_1c_ increase to be associated with glaucoma and refractive error for patients with diabetes, and in this pediatric cohort, we observed thinning of the GCL+IPL layer of −0.39 microns per 1% of HbA_1c_ increase. It is well known that youth with T1D have higher HbA_1c_ levels than other age groups with diabetes, and with a lifetime of diabetes ahead of them, GCL+IPL thinning may serve as an early marker of diabetic retinal neurodegeneration and should be studied longitudinally in the youth T1D population.^31^

This study also found an association between CGM TIR and outer retinal layer thickness. These findings support the consideration of future studies to investigate potential associations of CGM TIR and other CGM metrics, such as coefficient of variation, with retinal thickness measurement, especially as the use of CGMs increases in T1D.^32,33^

Thinning of the outer retinal layers may be due to decrease in choroidal perfusion, leading to outer retinal layer dysfunction or atrophy.^34^ Our prior work in a small cohort of youth with T1D and T2D who underwent OCT-angiography imaging suggests that there are changes in the choroidal complex that potentially precede DRD and may be related to this phenomenon.^23^

To our knowledge, there are limited data on racial differences in retinal thickness in general and in patients with T1D. Nevertheless, one study showed lower RNFL thickness in Black Americans aged 50 years or older compared with people of the same age with Chinese or Latin American ancestry, and a correlation between patients of African American race and lower foveal thickness compared with Hispanic and White patients.^35,36,37^ Our study similarly found Black race to be associated with lower RNFL thickness, GCL+IPL thickness, and outer retinal layer thickness, even when adjusting for differences in the total retinal layer thickness. However, in this cohort, race and HbA_1c_ were strongly correlated, with most Black participants having HbA_1c_ values of 8% or greater, which limits the ability to fully disentangle the independent effects of glycemic control and race. Future studies with more balanced cohorts could help elucidate these potential associations.

Although we did not find significant differences in retinal thickness by presence or absence of DRD or by severity of DRD, these analyses were limited by the small number of eyes with DRD and particularly moderate DRD (n = 10), resulting in imprecise estimates. There was a trend toward thickening of the layers in mild DRD and overall thinning in moderate DRD. Other studies have hypothesized that swelling of the GCL and outer retinal layers may precede thinning associated with neurodegeneration and later neurovascular disease.^38^ Instead, initial thickening may be an earlier marker of diabetic macular edema, which is characterized by retinal thickening as the capillary pericytes separate as a result of hyperglycemia. Other studies have demonstrated retinal layer thinning in the setting of DRD. A study of adults with T1D in Poland found mean, inferior, and nasal RNFL and superior and inferior GCL thickness to be lower in those with DRD than in those without DRD.^18^ Furthermore, a small observational study in Amsterdam with a mixed age group compared patients with T1D and minimal DRD to healthy controls and found GCL thickness to be lower in those with minimal DRD.^27^ It is possible that we did not see these differences due to our small sample size with mild and moderate DRD, and thus, larger pediatric studies will be important to further elucidate these trends.

Duration of diabetes is a risk factor for diabetic retinopathy, yet we did not identify any differences in RNFL, GCL+IPL, or outer retinal layer thickness by duration, while other studies have shown inverse associations between diabetes duration and RNFL thickness.^28,29^ This distinction from our study’s findings could be explained by our cohort’s shorter duration of diabetes in comparison to adult studies, again highlighting the importance of future longitudinal studies.

### Strengths and Limitations

Strengths of this study include its diverse cohort and being the largest sample of pediatric patients with T1D investigated for retinal thickness using OCT. This study also further analyzed possible correlations between insulin form, TIR, and retinal thickness—an important exploration in the current age of diabetes technology. Additionally, by adjusting for total retinal thickness in our analysis, we were able to account for thinning in the RNFL and in the GCL+IPL that were independent of possible demographic differences in total retinal thickness.^39^

Limitations of this study include the cohort’s small number of participants with moderate or greater DRD, which limited statistical power to evaluate associations across the spectrum of retinopathy and disease severity. It is also possible that there was subretinal or intraretinal fluid in the eyes of some participants, which may have affected measurements and limited our ability to assess their association with outcomes. Additionally, this study was conducted at a single institution and therefore may not be generalizable to other populations. Finally, several variables included in our study were related. Because few Black participants in our sample had low HbA_1c_ values, it is difficult to separate the effect of race from HbA_1c_ levels, even when controlling for both in the multivariable models. No adjustment was made for multiple comparisons, which increases the risk of type I error; therefore, these findings should be considered exploratory and hypothesis generating.

## Conclusions

In summary, this cohort study found higher HbA_1c_ was associated with neuroretinal and outer retinal layer thinning, even in the absence of clinically apparent DRD. Data on the trajectory of glycemic control across the lifespan show that youth with T1D have higher HbA_1c_ levels than adults, and even though there is no apparent DRD, these findings raise concern about early neuroretinal changes that may later develop into DRD. Additionally, these novel findings in a diverse cohort in the age of diabetes technologies offer novel contributions to the limited data on retinal thickness changes in youth with T1D. This study supports consideration of OCT measurements in pediatric populations with T1D if standardized reference values are established for diverse pediatric populations and if such measurements inform management in ways that improve clinically relevant outcomes. Future longitudinal studies including a greater number of participants with moderate or greater DRD will further elucidate the development of DRN and its potential role as a biomarker of retinal vascular disease.

## Notes

Supplement 1. eTable 1. Linear Mixed Effect Model for RNFL Thickness (µm) Accounting for Within-Subject Correlation eTable 2. Linear Mixed Effect Model for GCL+IPL Thickness (µm) Accounting for Within-Subject Correlation eTable 3. Linear Mixed Effect Model Total Retinal Thickness Without GCL+IPL+RNFL (µm) Accounting for Within-Subject Correlation eTable 4. Linear Mixed Effect Model for GCL+IPL Thickness (µm) Accounting for Within-Subject Correlation, Stratified: Black Participants Only eTable 5. Linear Mixed Effect Model for GCL+IPL Thickness (µm) Accounting for Within-Subject Correlation, Stratified: Non-Black Participants Only eTable 6. Total Retinal Thickness Without GCL+IPL+RNFL (µm) Accounting for Within-Subject Correlation, Stratified: Black Only Participants eTable 7. Total Retinal Thickness Without GCL+IPL+RNFL (µm) Accounting for Within-Subject Correlation, Stratified: Non-Black Participants Only eFigure 1. Optical Coherence Tomography eFigure 2. Diagram of Participant Enrollment, Exclusions, and Analysis eFigure 3. Association of Retinal Layers With HbA1C

Supplement 2. Data Sharing Statement
