# Supplementary material for: Neuroretinal Layer Thinning on OCT Imaging and Hemoglobin A1c in Youth With Type 1 Diabetes
Source: JAMA Ophthalmol. 2026 Jun 25:e261341. Online ahead of print. doi: 10.1001/jamaophthalmol.2026.1341 (PMC13306483; doi:10.1001/jamaophthalmol.2026.1341)
Supplement: Supplement 1. — eTable 1. Linear Mixed Effect Model for RNFL Thickness (µm) Accounting for Within-Subject Correlation eTable 2. Linear Mixed Effect Model for GCL+IPL Thickness (µm) Accounting for Within-Subject Correlation eTable 3. Linear Mixed Effect Model Total Retinal Thickness Without GCL+IPL+RNFL (µm) Accounting for Within-Subject Correlation eTable 4. Linear Mixed Effect Model for GCL+IPL Thickness (µm) Accounting for Within-Subject Correlation, Stratified: Black Participants Only eTable 5. Linear Mixed Effect Model for GCL+IPL Thickness (µm) Accounting for Within-Subject Correlation, Stratified: Non-Black Participants Only eTable 6. Total Retinal Thickness Without GCL+IPL+RNFL (µm) Accounting for Within-Subject Correlation, Stratified: Black Only Participants eTable 7. Total Retinal Thickness Without GCL+IPL+RNFL (µm) Accounting for Within-Subject Correlation, Stratified: Non-Black Participants Only eFigure 1. Optical Coherence Tomography eFigure 2. Diagram of Participant Enrollment, Exclusions, and Analysis eFigure 3. Association of Retinal Layers With HbA1C [file jamaophthalmol-e261341-s001.pdf]

Supplemental Online Content

Ramanujam S, Channa R, Liu TYA, et al. Neuroretinal layer thinning on OCT imaging and hemoglobin A_{1c} in youth with type 1 diabetes. *JAMA Ophthalmol*. Published online June 25, 2026. doi:10.1001/jamaophthalmol.2026.1341

eTable 1. Linear Mixed Effect Model for RNFL Thickness (μm) Accounting for Within-Subject Correlation

eTable 2. Linear Mixed Effect Model for GCL+IPL Thickness (μm) Accounting for Within-Subject Correlation

eTable 3. Linear Mixed Effect Model Total Retinal Thickness Without GCL+IPL+RNFL (μm) Accounting for Within-Subject Correlation

eTable 4. Linear Mixed Effect Model for GCL+IPL Thickness (μm) Accounting for Within-Subject Correlation, Stratified: Black Participants Only

eTable 5. Linear Mixed Effect Model for GCL+IPL Thickness (μm) Accounting for Within-Subject Correlation, Stratified: Non-Black Participants Only

eTable 6. Total Retinal Thickness Without GCL+IPL+RNFL (μm) Accounting for Within-Subject Correlation, Stratified: Black Only Participants

eTable 7. Total Retinal Thickness Without GCL+IPL+RNFL (μm) Accounting for Within-Subject Correlation, Stratified: Non-Black Participants Only

eFigure 1. Thinning of Ganglion Cell and Inner Plexiform Layer With High HbA1c

eFigure 2. Diagram of Participant Enrollment, Exclusions, and Analysis

eFigure 3. Association of Retinal Layers With HbA_{1c}

This supplemental material has been provided by the authors to give readers additional information about their work.

© 2026 Ramanujam S et al. *JAMA Ophthalmology*.

**eTable 1.** Linear Mixed Effect Model for RNFL Thickness (μm) Accounting for Within-Subject Correlation (n= 445* Eyes in Adjusted Model)					
		Unadjusted		Adjusted	
Effect		Estimate (95% CI)	*P* value	Estimate (95% CI)	*P* value
Intercept		-	-	11.22 (4.51,17.93)	.001
Female		-0.39 (-1.06,0.29)	.26	0.03 (-0.61,0.68)	.92
Race (ref=White)			.01		.48
	Asian	-2.01 (-5.48,1.47)	.26	-0.72 (-4.08,2.64)	.67
	Black	-1.47 (-2.30,-0.64)	.001	-0.86 (-1.8,0.09)	.07
	Hispanic	-0.65 (-2.44,1.14)	.48	-0.24 (-2.1,1.62)	.80
	Multi-Racial	-0.84 (-2.09,0.42)	.19	-0.46 (-1.68,0.75)	.45
Age		0.08 (-0.04,0.20)	.18	0.15 (0.02,0.28)	.03
Medicaid Insurance		-0.95 (-1.65,-0.26)	.008	-0.36 (-1.1,0.38)	.34
Duration of Diabetes		-0.01 (-0.10,0.08)	.86	-0.07 (-0.18,0.03)	.16
DRD Severity (ref=No Apparent Retinopathy)			.10		.15
	Mild DRD	0.18 (-0.43,0.79)	.56	0.22 (-0.4,0.83)	.49
	Moderate DRD	-1.64 (-3.27,-0.01)	.049	-1.41 (-3.04,0.22)	.09
% of Time in Target Range (70-180 mg/dL)		0.01 (-0.01,0.03)	.20	-0.004 (-0.02,0.01)	.64
Insulin Injections (ref=pump)		-0.62 (-1.47,0.24)	.15	-0.29 (-1.19,0.62)	.53
Total Retinal Thickness without RNFL		0.06 (0.04,0.09)	<.001	0.06 (0.03,0.08)	<.001
Downtown Site (ref = suburban)		0.08 (-0.60,0.75)	.83	0.48 (-0.22,1.17)	.18
*Includes only patients with CGM and Time in Target Range data.					

© 2026 Ramanujam S et al. *JAMA Ophthalmology*.

**eTable 2.** Linear Mixed Effect Model for GCL+IPL Thickness (µm) Accounting for Within-Subject Correlation (n= 445* Eyes in Adjusted Model)					
		Unadjusted		Adjusted	
Effect		Estimate (95% CI)	*P* value	Estimate (95% CI)	*P* value
Intercept		-	-	49.54 (39.64,59.43)	<.001
Female		-1.32 (-2.75,0.11)	.07	-0.75 (-2.14,0.63)	.28
Race (ref=White)			.18		.20
	Asian	-7.78 (-15.35,-0.21)	.04	-5.64 (-12.91,1.62)	.13
	Black	-1.17 (-2.98,0.63)	.20	1.85 (-0.19,3.9)	.07
	Hispanic	-2.06 (-5.93,1.8)	.29	0.69 (-3.32,4.7)	.74
	Multi-Racial	-0.23 (-2.95,2.49)	.87	1.16 (-1.48,3.8)	.39
Age		0.04 (-0.22,0.3)	.78	-0.13 (-0.41,0.16)	.38
Medicaid Insurance		-1.99 (-3.48,-0.5)	.009	-2.04 (-3.64,-0.44)	.01
Duration of Diabetes		0.17 (-0.02,0.37)	.09	0.13 (-0.09,0.35)	.25
DRD Severity (ref=No Apparent Retinopathy)			.02		.04
	Mild DRD	0.51 (-0.08,1.1)	.09	0.48 (-0.08,1.04)	.09
	Moderate DRD	-1.53 (-3.1,0.03)	.055	-1.16 (-2.64,0.32)	.13
% of Time in Target Range (70-180 mg/dL)		0.02 (-0.02,0.06)	.35	-0.01 (-0.05,0.03)	.61
Insulin Injections (ref=pump)		-1.69 (-3.51,0.13)	.07	-1.53 (-3.5,0.44)	.13
Total Retinal Thickness without GCL+IPL+RNFL		0.17 (0.13, 0.22)	<.001	0.17 (0.12,0.22)	<.001
Downtown Site (ref = suburban)		-0.74 (-2.17,0.69)	.31	0.06 (-1.44,1.57)	.93
*Includes only patients with CGM and Time in Target Range data.					

© 2026 Ramanujam S et al. *JAMA Ophthalmology*.

**eTable 3.** Linear Mixed Effect Model Total Retinal Thickness Without GCL+IPL+RNFL (µm) Accounting for Within-Subject Correlation (n= 445* Eyes)					
		Unadjusted		Adjusted	
Effect		Estimate (95% CI)	*P* value	Estimate (95% CI)	*P* value
Intercept		-	-	176.27 (167.49,185.06)	<.001
Female		-3.86 (-6.44,-1.28)	.004	-2.82 (-5.23,-0.41)	.02
Race (ref=White)			<.001		<.001
	Asian	-13.23 (-26.05,-0.41)	.04	-10.53 (-23.19,2.13)	.10
	Black	-9.55 (-12.6,-6.49)	<.001	-8.67 (-12.16,-5.18)	<.001
	Hispanic	-4.53 (-11.08,2.02)	.17	-4.44 (-11.45,2.56)	.21
	Multi-Racial	-4.88 (-9.49,-0.27)	.04	-4.18 (-8.79,0.42)	.07
Age		0.20 (-0.28,0.67)	.41	-0.001 (-0.5,0.5)	.99
Medicaid Insurance		-2.85 (-5.59,-0.11)	.04	0.16 (-2.64,2.96)	.91
Duration of Diabetes		0.43 (0.07,0.78)	.02	0.36 (-0.03,0.74)	.07
DRD Severity (ref=No Apparent Retinopathy)			.37		.42
	Mild DRD	0.09 (-1.1,1.28)	.88	0.23 (-0.96,1.42)	.71
	Moderate DRD	-2.16 (-5.32,1)	.18	-1.88 (-5.03,1.28)	.24
% of Time in Target Range (70-180 mg/dL)		0.12 (0.05,0.19)	.001	0.08 (0.01,0.15)	.03
Insulin Injections (ref=pump)		-1.37 (-4.71,1.97)	.42	3.14 (-0.29,6.58)	.07
Downtown Site (ref = suburban)		-3.72 (-6.29,-1.15)	.005	-1.26 (-3.89,1.37)	.35
*Includes only patients with CGM and Time in Target Range data.					

© 2026 Ramanujam S et al. *JAMA Ophthalmology*.

**eTable 4.** Linear Mixed Effect Model for GCL+IPL Thickness (µm) Accounting for Within-Subject Correlation, Stratified: Black Participants Only (n= 182 Eyes)				
	Unadjusted		Adjusted	
Effect	Estimate (95% CI)	*P* value	Estimate (95% CI)	*P* value
Intercept	-	-	68.55 (49.33, 87.77)	<.001
Female	-2.48 (-4.85, -0.12)	.04	-2.39 (-4.52, -0.25)	.03
Age	0.01 (-0.41, 0.43)	.96	-0.27 (-0.68, 0.14)	.19
Medicaid Insurance	-1.62 (-4.11, 0.88)	.20	-1.28 (-3.46, 0.89)	.24
Duration of Diabetes	0.36 (0.04, 0.67)	.03	0.38 (0.08, 0.68)	.01
DRD Severity (ref=No Apparent Retinopathy)		.29		.28
Mild DRD	0.75 (-0.23, 1.73)	.13	0.8 (-0.19, 1.8)	.11
Moderate DRD	0.03 (-1.95, 2.01)	.98	0.31 (-1.71, 2.33)	.76
Hemoglobin A1c (average over 5 most recent visits)	-0.92 (-1.45, -0.40)	.001	-0.95 (-1.5, -0.41)	.001
Insulin Injections (ref=pump)	-1.13 (-3.54, 1.29)	.36	0.96 (-1.34, 3.26)	.41
Total Retinal Thickness without GCL+IPL+RNFL	0.18 (0.09, 0.26)	<.001	0.12 (0.03, 0.2)	.008
Downtown Site (ref = suburban)	-1.32 (-3.84, 1.19)	.30	0.07 (-2.38, 2.51)	.96

© 2026 Ramanujam S et al. *JAMA Ophthalmology*.

**eTable 5.** Linear Mixed Effect Model for GCL+IPL Thickness (μm) Accounting for Within-Subject Correlation, Stratified: Non-Black Participants Only (n=393 Eyes)					
		Unadjusted		Adjusted	
Effect		Estimate (95% CI)	*P* value	Estimate (95% CI)	*P* value
Intercept		-	-	44.15 (32.98,55.32)	<.001
Female		-0.93 (-2.61,0.76)	.28	-0.76 (-2.34,0.82)	.34
Age		0.04 (-0.27,0.35)	.79	-0.03 (-0.36,0.31)	.86
Medicaid Insurance		-1.88 (-3.84,0.07)	.06	-1.27 (-3.19,0.65)	.19
Duration of Diabetes		0.06 (-0.17,0.29)	.61	0.02 (-0.24,0.27)	.90
DRD Severity (ref=No Apparent Retinopathy)			.012		.02
	Mild DRD	0.12 (-0.55,0.78)	.73	0.19 (-0.44,0.81)	.56
	Moderate DRD	-3.07 (-5.14,-1.00)	.004	-2.67 (-4.61,-0.73)	.007
Hemoglobin A1c (average over 5 most recent visits)		-0.33 (-0.86,0.21)	.23	-0.04 (-0.58,0.49)	.87
Insulin Injections (ref=pump)		-2.27 (-4.52,-0.02)	.048	-2.02 (-4.34,0.3)	.09
Total Retinal Thickness without GCL+IPL+RNFL		0.20 (0.15, 0.25)	<.001	0.19 (0.14,0.24)	<.001
Downtown Site (ref = suburban)		-0.45 (-2.14,1.24)	.60	0.21 (-1.44,1.87)	.80

© 2026 Ramanujam S et al. *JAMA Ophthalmology*.

**eTable 6.** Total Retinal Thickness Without GCL+IPL+RNFL (µm) Accounting for Within-Subject Correlation, Stratified: Black Only Participants (n=182 Eyes)				
	Unadjusted		Adjusted	
Effect	Estimate (95% CI)	*P* value	Estimate (95% CI)	*P* value
Intercept	-	-	197.67 (179.18,216.17)	<.001
Female	-5.43 (-9.72,-1.14)	.01	-6.38 (-10.6,-2.16)	.004
Age	0.08 (-0.69,0.85)	.84	-0.46 (-1.29,0.37)	.27
Medicaid Insurance	-1.95 (-6.54,2.64)	.40	-1.3 (-5.74,3.13)	.56
Duration of Diabetes	0.35 (-0.23,0.93)	.24	0.3 (-0.31,0.92)	.32
DRD Severity (ref=No Apparent Retinopathy)		.92		.86
Mild DRD	0.26 (-1.20,1.72)	.72	0.37 (-1.09,1.83)	.62
Moderate DRD	0.40 (-2.57,3.37)	.79	0.59 (-2.39,3.56)	.70
Hemoglobin A1c (average over 5 most recent visits)	-1.20 (-2.20,-0.21)	.02	-1.36 (-2.44,-0.28)	.02
Insulin Injections (ref=pump)	-1.69 (-6.12,2.74)	.45	0.17 (-4.53,4.87)	.94
Downtown Site (ref = suburban)	-3.84 (-8.41,0.73)	.10	-3.41 (-8.38,1.56)	.18

© 2026 Ramanujam S et al. *JAMA Ophthalmology*.

**eTable 7.** Total Retinal Thickness Without GCL+IPL+RNFL (µm) Accounting for Within-Subject Correlation, Stratified: Non-Black Participants Only (n=393 Eyes)					
		Unadjusted		Adjusted	
Effect		Estimate (95% CI)	*P* value	Estimate (95% CI)	*P* value
Intercept		-	-	180.94 (169.37,192.52)	<.001
Female		-1.89 (-4.59,0.81)	.17	-1.91 (-4.64,0.81)	.17
Age		0.46 (-0.04,0.96)	.07	0.24 (-0.34,0.82)	.41
Medicaid Insurance		-1.00 (-4.17,2.16)	.53	-0.95 (-4.26,2.37)	.57
Duration of Diabetes		0.31 (-0.06,0.68)	.10	0.21 (-0.23,0.65)	.34
DRD Severity (ref=No Apparent Retinopathy)			.68		.70
	Mild DRD	-0.29 (-1.71,1.13)	.69	-0.28 (-1.7,1.14)	.70
	Moderate DRD	-1.85 (-6.27,2.57)	.41	-1.79 (-6.21,2.64)	.43
Hemoglobin A1c (average over 5 most recent visits)		-0.35(-1.21,0.51)	.42	-0.47 (-1.38,0.45)	.32
Insulin Injections (ref=pump)		0.54 (-3.11,4.19)	.77	1.31 (-2.7,5.31)	.52
Downtown Site (ref = suburban)		-2.58 (-5.27,0.11)	.06	-1.68 (-4.54,1.18)	.25

© 2026 Ramanujam S et al. *JAMA Ophthalmology*.

eFigure 1. Thinning of Ganglion Cell and Inner Plexiform Layer With High HbA1c

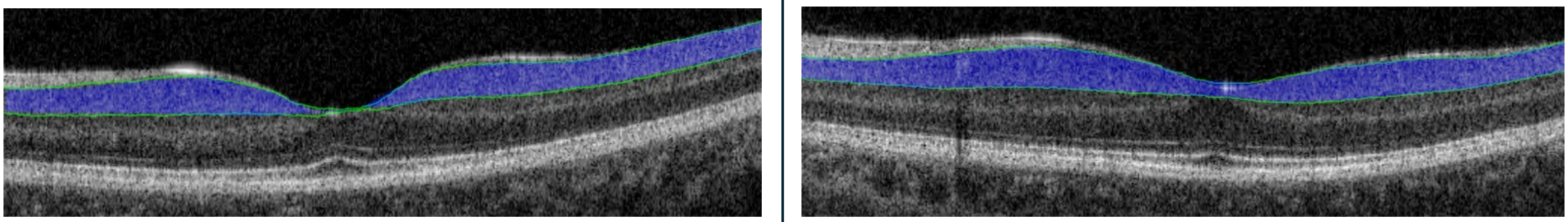

GCL-IPL thickness = 79.8 µ-meters with HbA1c of 6.9%

GCL-IPL thickness = 68.7 µ-meters with HbA1c of 11%

GCL-IPL, ganglion cell layer - inner plexiform layer.

© 2026 Ramanujam S et al. *JAMA Ophthalmology*.

eFigure 2. Diagram of Participant Enrollment, Exclusions, and Analysis

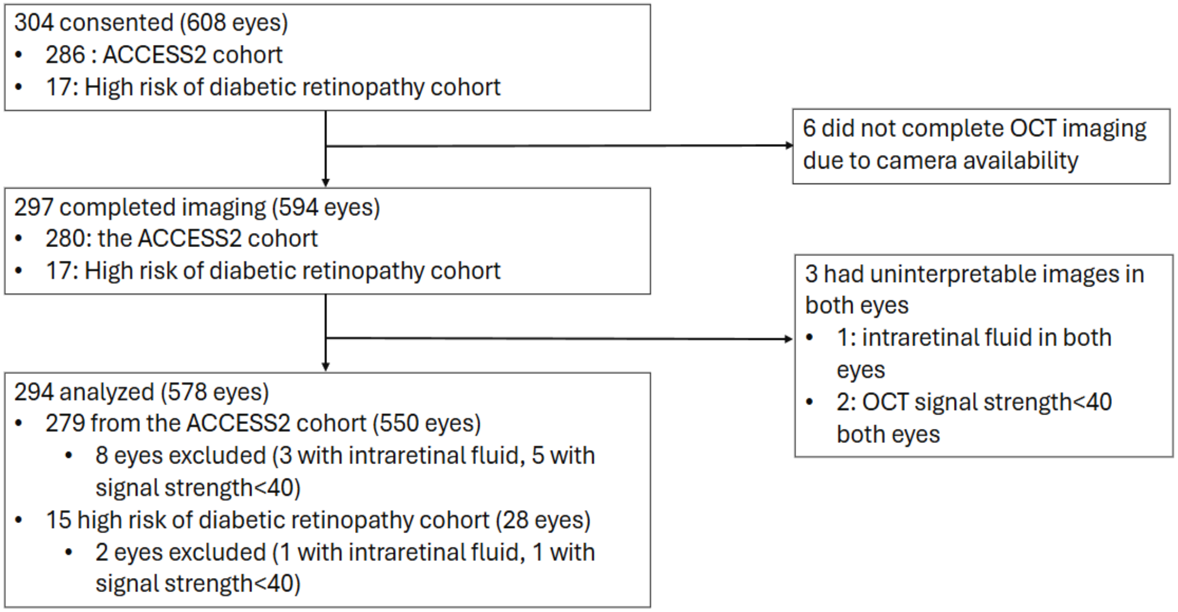

© 2026 Ramanujam S et al. *JAMA Ophthalmology*.

eFigure 3. Association of Retinal Layers With HbA_{1c}

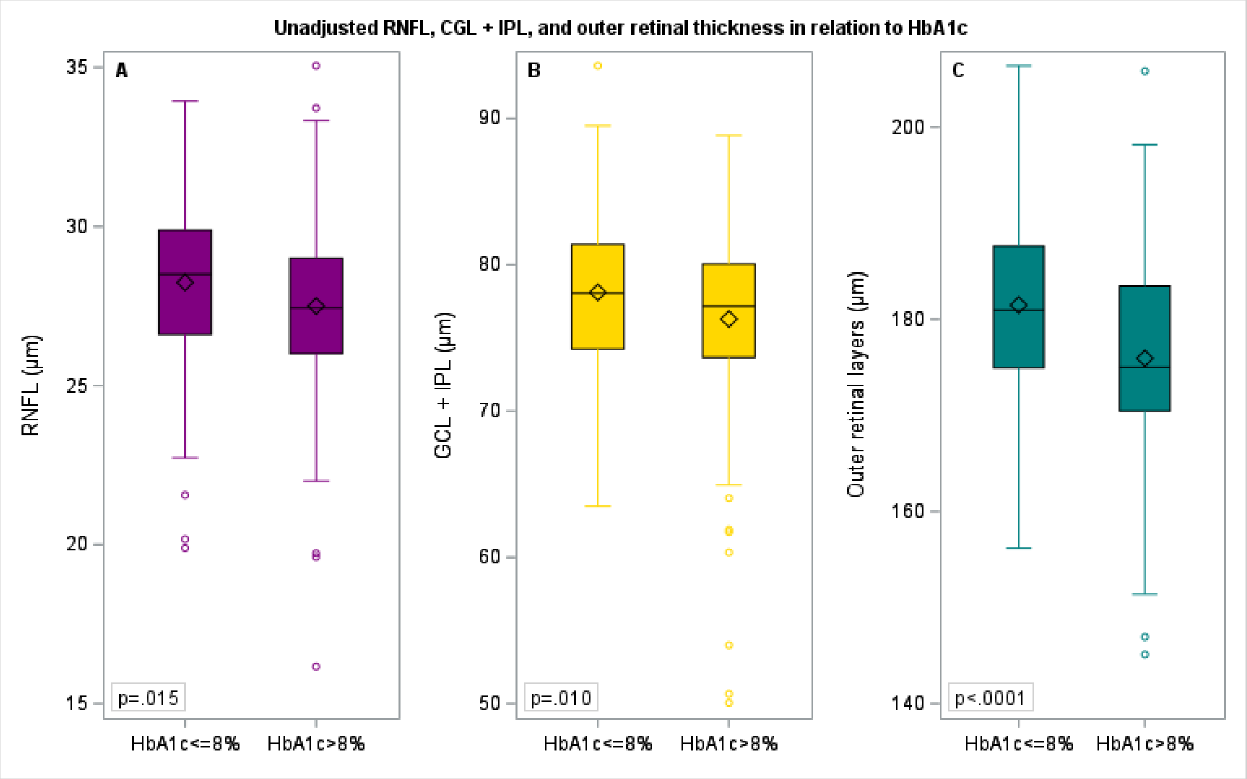

Caption: Plots show the average measures of two eyes. Boxplot: the line inside the box shows the median and the diamond indicates the mean. The box spans the first to third quartiles (Q1–Q3). Whiskers extend to the most extreme values within 1.5×IQR, and points beyond the whiskers are plotted as outliers. P values calculated using two-sample T tests and average measurements from both eyes

© 2026 Ramanujam S et al. *JAMA Ophthalmology*.
