# Supplementary material for: Neuroretinal Layer Thinning on OCT Imaging and Hemoglobin A1c in Youth With Type 1 Diabetes
Source: JAMA Ophthalmol. 2026 Jun 25:e261341. Online ahead of print. doi: 10.1001/jamaophthalmol.2026.1341 (PMC13306483; doi:10.1001/jamaophthalmol.2026.1341)
Supplement: Supplement 2. — Data Sharing Statement [file jamaophthalmol-e261341-s002.pdf]

Data Sharing Statement

Ramanujam. Neuroretinal Layer Thinning on OCT Imaging and Hemoglobin A_{1c} in Youth With Type 1 Diabetes. *JAMA Ophthalmol*. Published June 25, 2026.
doi:10.1001/jamaophthalmol.2026.1341

Data
Data available: Yes
Data types: Deidentified participant data
How to access data: Anyone who needs the data must formally request it and request should be directed to Dr.Risa Wolf - e-mail address: rwolf@jhu.edu
When available: With publication

Supporting Documents
Document types: None

Additional Information
Who can access the data: researchers whose proposed use of the data has been approved
Types of analyses: for a specified purpose
Mechanisms of data availability: with a signed data access agreement
Any additional restrictions: None
